# The dataset describes: Phenotypic changes induced by cholesterol loading in smooth muscle cells isolated from the aortae of C57BL/6 mice

**DOI:** 10.1016/j.dib.2017.11.050

**Published:** 2017-11-20

**Authors:** Silvia Castiglioni, Matteo Monti, Giuditta Ainis Buscherini, Lorenzo Arnaboldi, Monica Canavesi, Alberto Corsini, Stefano Bellosta

**Affiliations:** a Department of Pharmacological and Biomolecular Sciences, Università degli Studi di Milano, Milan, Italy; b IRCCS MultiMedica, Milan, Italy

**Keywords:** ABCA1, Cholesterol, HDL3, Phenotypic switch, Smooth muscle cells

## Abstract

The data presented in this article is related to the research article entitled “*ABCA1 and HDL_3_ are Required to Modulate Smooth Muscle Cells Phenotypic Switch after Cholesterol Loading***”** (Castiglioni et al., 2017) [1]. This data describes the characterization of the phenotypic changes induced by cholesterol loading in smooth muscle cells (SMCs) isolated from the aortae of C57BL/6 mice. Upon cholesterol loading, there is a significant and concentration-dependent decrease in the expression of *Acta2* and a parallel increase in *Mac-2,* and ATP binding cassette (ABC) transporters *Abca1* and *Abcg1*. Cholesterol incubation causes the transformation of SMCs into foam cells with a 3-fold increase in cellular total cholesterol content and a 2.5-fold stimulation of the activity of the esterifying enzyme Acyl-CoA:cholesterol acyltransferase (ACAT). The addition of the same amount of cholesterol, either dissolved in ethanol or as lipoprotein cholesterol (AcLDL or native LDL) only slightly induces the activity of the enzyme ACAT, and does not cause the accumulation of lipid droplets into SMCs. We describe also the knock down of ABCA1 expression by siRNA treatment in mouse smooth muscle cells.

**Specifications Table**

**Table:** 

Subject area	*Cell Biology*
More specific subject area	*Mechanism of atherosclerosis*
Type of data	*Graphs, figures*
How data was acquired	*Microscope, Real time PCR (Applied Biosystem ABI7000), western blot analysis, ACAT enzyme activity, gas liquid chromatography*
Data format	*Analyzed*
Experimental factors	*Smooth muscle cells were isolated from the aorta of C56BL/6, wild type or ABCA1 knock out mice*
Experimental features	*Real time PCR and western blot analyses were performed on mouse smooth muscle cells previously incubated with cholesterol complexed with methyl-beta-cyclodextrin*
Data source location	*Milan, Italy*
Data accessibility	*Data is within this article.*

**Value of the data**

• This data describes the cholesterol-induced phenotypic changes in murine smooth muscle cells.
• Cholesterol loading downregulates the expression of *smooth muscle cell* markers, and increases the expression of inflammation-related genes. In addition, cholesterol transforms smooth muscle cells into foam cells and affects lipid metabolism.
• The present data gives insights on how deliver cholesterol to metabolically active intracellular lipid pools, independently of lipoprotein receptor pathway.

## 1 Data

**Fig. 1:**
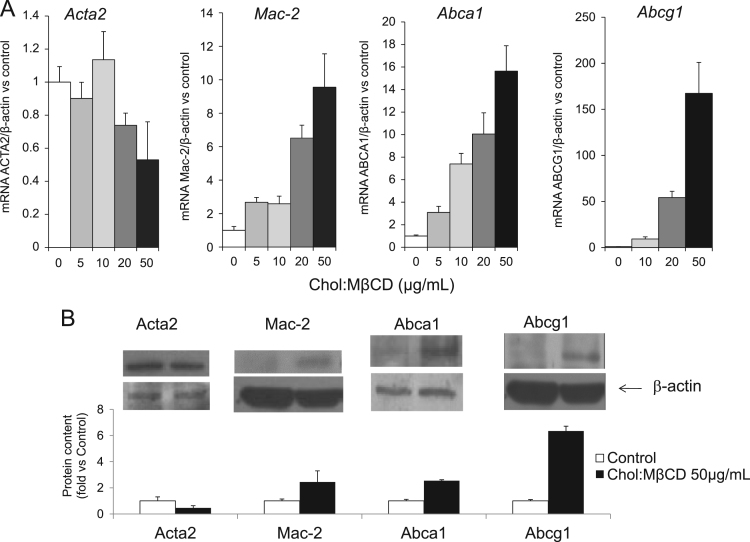
Effects of cholesterol-loading on mRNA and protein expression in SMC. SMCs isolated from C57BL/6 mice were treated with increasing concentrations of Chol:MβCD in DMEM 0.2% EFAF. After 48 h, total mRNA and proteins were extracted and subjected to qRT-PCR (A) or Western blot analysis (B) for Acta2, Mac-2, Abca1, and Abcg1, as described in Materials and Methods. Data are expressed as mean ± SD of three experiments performed in triplicates.

**Fig. 2:**
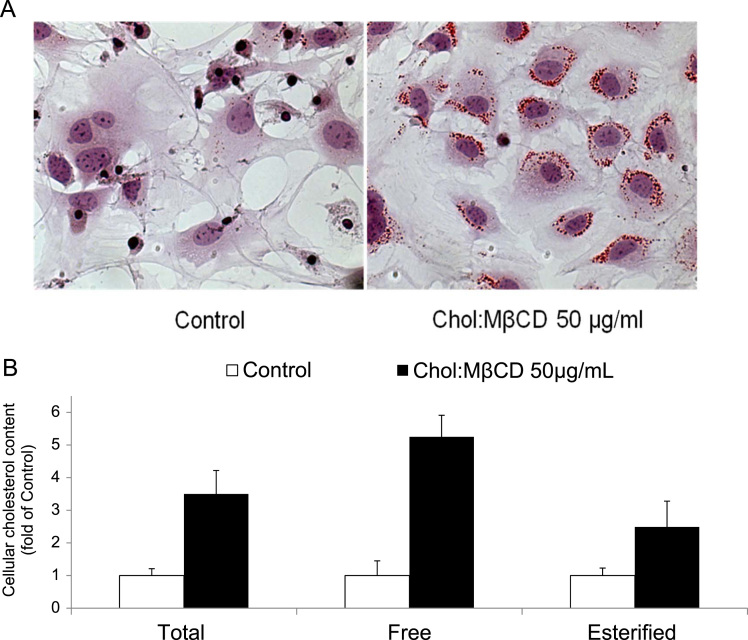
Cholesterol loading induces the accumulation of lipids in C57BL/6 SMCs. (A) Mouse SMCs were treated with Chol:MβCD (50 μg/mL) in DMEM 0.2% EFAF. After 48 h cells were fixed in 4% paraformaldehyde, stained with Oil Red O and light microscopic images acquired (original magnification X80). (B) In another set of cells, cellular lipids were extracted and total, free and esterified cholesterol measured as described in Materials and Methods (Control cells: total cholesterol 41.3 μg/mg cell prot, free cholesterol 30.1 μg/mg cell prot, esterified cholesterol 11.2 μg/mg cell prot.). Data are expressed as mean ± SD of three experiments performed in triplicates.

**Fig. 3:**
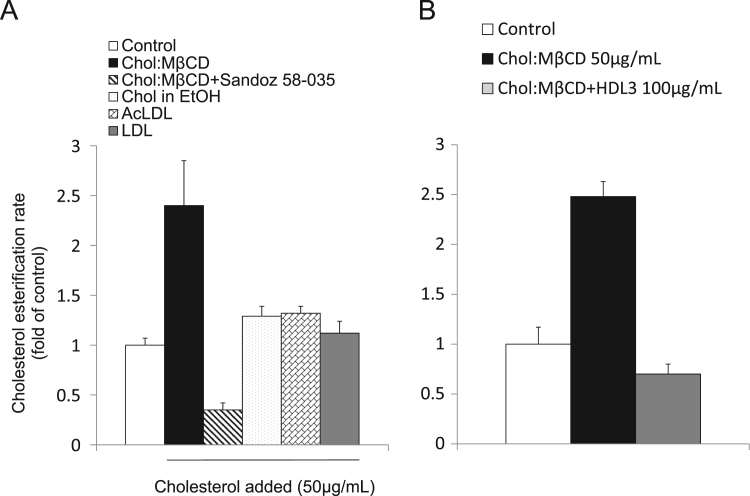
Effect of lipoprotein or non-lipoprotein cholesterol on cholesterol esterification in murine SMCs. (A) C57BL/6 mice SMCs were treated for 48 h with cholesterol (50 μg/mL) added as Chol:MβCD, dissolved in ethanol or as lipoproteins (AcLDL or LDL). In the last 2h of incubation, ^14^C-oleic acid was added and cholesterol esterification measured as described in Materials and Methods. (B) SMCs were incubated with Chol:MβCD (50 μg/mL) in presence or absence of HDL_3_ (100 μg/mL). Data are expressed as mean ± SD of two experiments performed in triplicates.

This dataset extends and completes the data presented in the recently published article [1]. Cholesterol loading of smooth muscle cells (SMCs) isolated from the aortae of C57BL/6 mice causes a significant and concentration-dependent decrease in *Acta2* mRNA levels (Fig. 1A) and an increase in *Mac-2, Abca1* and *Abcg1* mRNA (up to 10-fold, 16-fold and 160-fold, respectively). The effects on mRNAs are confirmed by western blot analysis (Fig. 1B). Cholesterol incubation causes the transformation of SMCs into foam cells, with intracellular accumulation of lipid droplets (Oil Red O staining; Fig. 2A). There is a 3-fold increase in cellular total cholesterol content, due to the accumulation of both free and esterified cholesterol (Fig. 2B). The increased cellular esterified cholesterol content is caused by a 2.5-fold stimulation of the activity of the esterifying enzyme ACAT that is completely blocked by the addition of Sandoz 58-035, a specific ACAT inhibitor (Fig. 3A). The addition of the same amount of cholesterol, either dissolved in ethanol or as lipoprotein cholesterol (native low-density lipoprotein (LDL) or Acetylated LDL) slightly induces the activity of the ACAT enzyme (Fig. 3A), and does not cause the accumulation of lipid droplets into SMCs (data not shown). The cholesterol delivered is available for downloading in the presence of HDL_3_ (Fig. 3B).

**Fig. 4:**
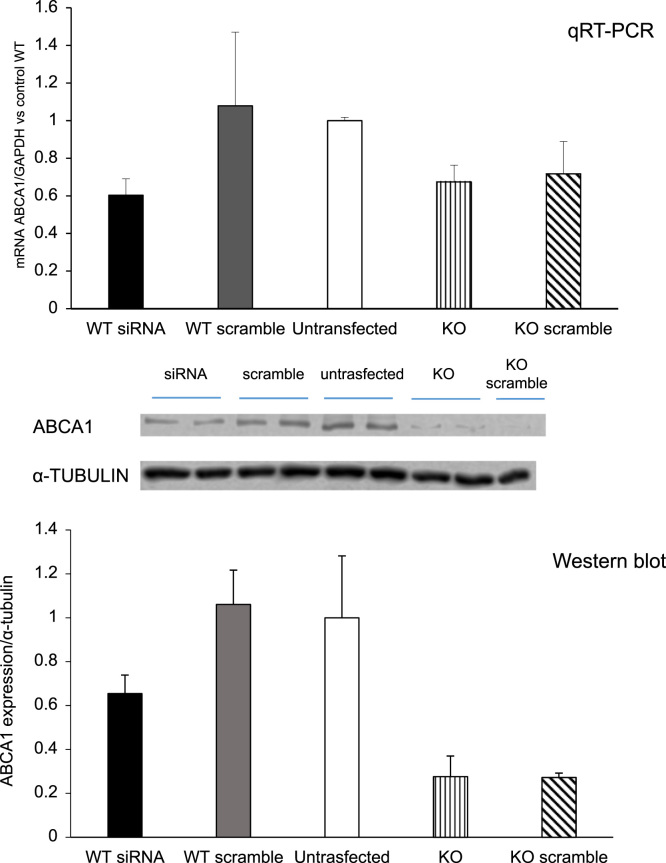
Effect of *Abca*1 siRNA treatment on Abca1 expression. WT SMCs were treated for 24 h with 10 nM *Abca*1 siRNA or a nonsense strand of siRNA (scramble). Then cells were treated for 48 h with Chol:MβCD (50 μg/mL) in DMEM 0.2% EFAF in presence or absence of HDL_3_ (100 μg/mL). Total mRNA and protein were extracted and subjected to qRT-PCR analysis or western blot analysis for Abca1 as described in Material and Methods. Data are expressed as mean ± SD of three experiments performed in triplicates.

As shown in Fig. 4, the incubation of WT SMC with *Abca1* siRNA reduces Abca1 expression to the levels observed in Abca1 KO cells, as measured by either qRT-PCR or western blot analysis (Fig. 4).

## 2 Experimental design, materials and methods

### 2.1 Cell cultures

SMCs were isolated from the intimal-medial layer of aortae of littermate Abca1 WT and KO mice of both sexes (The Jackson Lab, Bar Harbor, ME, USA). The mice, originally on a DBA background, have been backcrossed into C57BL/6 mice for at least nine times. All mice were kept in accordance with guidelines from Directive 2010/63/EU of the European Parliament on the protection of animals used for scientific purposes and the Italian Ministry of Health and the local University of Milan ethics committee approved the protocol. Mice were anesthetized with 2% isoflurane and killed by cervical dislocation. Aorta was rapidly dissected from the aortic root to the iliac bifurcation and SMCs prepared according to the procedure described by Ross [2].

Cells were grown in Dulbecco's modified Eagle's medium (DMEM) supplemented with 20% FCS. The medium was changed every three days. SMC lineage was confirmed by the presence of immunoreactivity for Acta2 in > 99% of the cells. The experiments have been performed using 8 cell lines isolated from different mice of both genotypes. Cells were used between the 4th and 10th passage.

### 2.2 HDL_3_ isolation

HDL_3_ were isolated from the plasma of healthy normolipidemic volunteers by sequential preparative ultracentrifugation [3].

### 2.3 Cell treatment

Cholesterol was delivered to cells by using a Chol:MβCD complex as ‘‘water-soluble cholesterol’’ containing ≈ 50 mg of cholesterol/g solid (molar ratio, 1:6 cholesterol/MβCD, Sigma-Aldrich) [4], [5]. All treatment concentrations involving Chol:MβCD were based on cholesterol weight. SMCs were incubated with DMEM supplemented with 0.2% Essential Fatty Acid Free albumin alone or containing Chol:MβCD (50 µg/ml) or HDL_3_ (100 µg/ml) or apoAI (35 µg/ml) for 48 h.

### 2.4 Oil Red O staining

Cholesterol-loaded SMCs were fixed for 30 min with 4% paraformaldehyde solution in PBS, stained with Oil Red O for 4 h and counterstained with hematoxylin for other 5 min [6]. Cells were examined with a light microscope (X80) and pictures of representative fields were taken.

### 2.5 Cholesterol esterification

After cholesterol loading, ^14^C-oleic acid was added and cholesterol esterification measured following the incorporation of labelled oleate into cellular cholesteryl esters [7].

### 2.6 Extraction and analysis of cellular cholesterol

Cell lipids were extracted in hexane/isopropyl alcohol (3:2) containing BHT 0.01%. Free- and esterified cholesterol were separated by HPTLC plates and quantified on a DANI 1000 gas liquid chromatographer [8]. Results were normalized by protein content.

### 2.7 mRNA and miRNA levels measurement

Total RNA was extracted by using the TRIZOL solution (Invitrogen) according to manufacturer's protocol and reverse transcribed (Thermo Scientific). SYBR Green-based real time PCR was used to measure mRNA or miRNA levels [9]. The primers used for specific mouse genes are listed in Table 1.

**Table 1:** Sequences of mouse primers.

**Gene name**	**Sequences**	**Gene name**	**Sequences**
***Abca1***	FW 5′-AAAACCGCAGACATCCTTCAG-3′	***Mac-2***	FW 5′-TGGGCACAGTGAAACCCAAC-3′
	RV 5′-CATACCGAAACTCGTTCACCC-3′		RV 5′-TCCTGCTTCGTGTTACACACA-3′
***Abcg1***	FW 5′-CCTTATCAATGGAATGCCCCG-3′	***Myocd***	FW 5′- AAGGTCCATTCCAACTGCTC-3′
	RV 5′-CTGCCTTCATCCTTCTCCTG-3′		RV 5′-CCATCTCTACTGCTGTCATCC-3′
***Acta2***	FW 5′-GTCCCAGACATCAGGGAGTAA-3′	***Klf4***	FW 5′-CTTTCCTGCCAGACCAGATG-3′
	RV 5′-TCGGATACTTCAGCGTCAGGA-3′		RV 5′-GGTTTCTCGCCTGTGTGAGT-3′
***Calponin***	FW 5′-TTGAGAGAAGGCAGGAACATC-3′		
	RV 5′-GTACCCAGTTTGGGATCATAGAG-3′		

### 2.8 siRNA treatment

Wild type SMCs were transfected for 24 h with *Abca1* siRNA (ON-TARGETplus SMARTpool Mouse Abca1, Dharmacon) or a nonsense strand of siRNA (scramble, ON-TARGETplus Non-Targeting pool, Dharmacon), using INTERFERin siRNA transfection reagent (Polyplus). Then, cells were treated for 48 hours with Chol:MβCD (50 μg/mL) in presence or absence of HDL_3_ (100 μg/mL). Total mRNA was extracted and subjected to qRT-PCR analysis.

### 2.9 Western blot

Mouse SMCs were processed for Western blotting as described [9]. The different proteins were detected using the following primary antibodies: Abca1 (1:1000; Abcam), Abcg1 (1:500; Novus Biologicals), Acta2 (1:10,000; Abcam), Mac-2 (1:1000: Abcam), β-actin (1:5000; Sigma-Aldrich), α-tubulin (1.5000; Sigma-Aldrich). Quantification was performed by densitometric analysis using the Image Studio Lite software from Li-Cor Bioscience.

### 2.10 Statistical analysis

Data were expressed as means ± SD. All experiments were repeated at least four-five times in triplicates.
