# Supplementary material for: The dataset describes: Phenotypic changes induced by cholesterol loading in smooth muscle cells isolated from the aortae of C57BL/6 mice
Source: Data Brief. 2017 Nov 20;16:334–40. doi: 10.1016/j.dib.2017.11.050 (PMC5723262; doi:10.1016/j.dib.2017.11.050)
Supplement: Supplementary file 1 — Transparency document [file mmc1.pdf]

UNIVERSITÀ DEGLI STUDI DI MILANO
DIPARTIMENTO DI ONCOLOGIA ED EMATO-ONCOLOGIA

Milan, October 10th, 2017

Dr **Marina Irène Ostankovitch** Ph.D
Senior Scientific Editor

Ref: Ms DIB-D-17-00901

Conflict of interest form

Dear Dr. Ostankovitch,

On behalf of my co-authors of the manuscript entitled "***The dataset describes: Phenotypic changes induced by cholesterol loading in smooth muscle cells isolated from the aortae of C57BL/6 mice***" by Silvia Castiglioni, Matteo Monti, Giuditta Ainis Buscherini, Lorenzo Arnaboldi, Monica Canavesi, Alberto Corsini, and myself, submitted to be considered for publication on *Data in Brief*

I hereby declare that none of the authors has any conflicts of interests.

Sincerely,

Stefano Bellosta

Stefano Bellosta, Ph.D.
Assistant Professor of Pharmacology,
Department of Pharmacological and Biomolecular Sciences, University of Milan,
Via Balzaretti 9, 20133 Milan, Italy
Phone: +39 02 5031 8392; Fax: +39 02 5031 8284
Email: Stefano.Bellosta@unimi.it
Mobile: +39 3394314292

Stefano Bellosta, Ph.D. Università degli Studi di Milano -Dipartimento di Oncologia ed emato-oncologia
Via Festa del Perdono, n°7 – CAP 20122 Milano, Italy. Tel 02 50312902 – Fax 02 50312904
Via Balzaretti 9, 20133 Milano. Tel +390250318392 – Fax +39 02 50318284 – Cell +393394314292
E-mai: stefano.bellosta@unimi.it
